# Neurodegenerative Interplay of Cardiovascular Autonomic Dysregulation and the Retina in Early Multiple Sclerosis

**DOI:** 10.3389/fneur.2019.00507

**Published:** 2019-05-15

**Authors:** Sigrid A. de Rodez Benavent, Gro O. Nygaard, Kristian B. Nilsen, Lars Etholm, Piotr Sowa, Marte Wendel-Haga, Hanne F. Harbo, Liv Drolsum, Bruno Laeng, Emilia Kerty, Elisabeth G. Celius

**Affiliations:** ^1^Department of Ophthalmology, Oslo University Hospital, Oslo, Norway; ^2^Faculty of Medicine, Institute of Clinical Medicine, University of Oslo, Oslo, Norway; ^3^Department of Neurology, Oslo University Hospital, Oslo, Norway; ^4^Section for Clinical Neurophysiology, Department of Neurology, Oslo University Hospital, Oslo, Norway; ^5^Department of Neuromedicine and Movement Science, Faculty of Medicine and Health Sciences, Norwegian University of Science and Technology, Trondheim, Norway; ^6^Section for Clinical Neurophysiology, Department of Neurology, Oslo University Hospital, Oslo, Norway; ^7^Division of Radiology and Nuclear Medicine, Oslo University Hospital, Oslo, Norway; ^8^Department of Neurology, Telemark Hospital, Skien, Norway; ^9^Department of Psychology, University of Oslo, Oslo, Norway

**Keywords:** multiple sclerosis, neurodegeneration, autonomic nervous system, optical coherence tomography, pupillometry, fatigue, postural orthostatic tachycardia

## Abstract

**Introduction:** Autonomic nervous system (ANS) symptoms are prevalent in multiple sclerosis (MS) as is neurodegeneration. Our aim was to explore the occurrence of ANS symptoms and retinal neurodegeneration in a newly diagnosed MS population with tools available in a clinical setting.

**Methods:** Forty-three MS patients and 44 healthy controls took part in the study. We employed a bedside cardiovascular ANS test battery together with classical pupillometry, optical coherence tomography (OCT) evaluation of retinal neurodegeneration in eyes without previous optic neuritis (MSNON) and patients' self-report forms on fatigue, orthostatic and ANS symptoms.

**Results:** Half of the patients presented with ANS symptoms and a high level of fatigue. There was a significant difference in ganglion cell layer thickness (mean GCIPL) evaluated by OCT in MSNON compared to healthy control eyes. We found a negative linearity of mean GCIPL on group level with increasing disease duration. Three patients fulfilled the criteria of postural orthostatic tachycardia syndrome (POTS).

**Conclusion:** Our results demonstrate retinal neurodegeneration in MSNON, a high frequency of fatigue and a high prevalence of ANS symptoms in newly diagnosed patients. Whether neurodegeneration precedes ANS dysfunction or vice versa is still open to debate, but as unveiled by the presence of POTS in this MS population, differences in stress-response regulation add to the understanding of variation in onset-time of ANS dysfunction in early MS.

## Introduction

Multiple sclerosis (MS) is a chronic disease characterized by inflammation and neurodegeneration in the central nervous system (CNS). The disease course is highly variable and the cause is unknown (1). MS patients often present symptoms of autonomic dysfunction (2, 3). Autonomic dysfunction and neurodegeneration seem to be linked in MS (2). Correlations between autonomic symptoms and specific parts of the CNS exist (4, 5), but studying autonomic dysfunction in MS is complicated because of the plasticity of this system and its' widespread activation. This complexity may in part be the reason why there is no common standardized autonomic test battery for MS.

The visual system offers a possibility to observe both the autonomic nervous system (ANS) and ongoing neurodegeneration utilizing pupillometry and optical coherence tomography (OCT). Classic study design, measuring the pupillary light reflex (PLR) with defined indices for parasympathetic and sympathetic function (6–9), has yielded changes in ANS function in MS patients (10–12). A link between PLR values and retinal architecture in MS patients has also been showed in advanced stages of the disease (13). OCT performs optical slicing of the retinal layers evaluating both the ganglion nerve cell layer and their peripapillar axons before leaving the eye and continuing as the optic nerve (14). OCT studies in MS have shown neurodegeneration in eyes with and without prior optic neuritis (MSNON) (15, 16). In order to examine the presence of ongoing neurodegeneration in early MS, we chose to examine MSNON.

MS may affect the ANS not only through neurodegeneration, but also by modulating the sympathetic nervous system peripherally through catecholamine release from inflammatory lesions, or by inflammatory induced expression of catecholamine receptors (17). Symptoms of orthostatic intolerance are frequent in MS (18–20) and may be explained as a result of inflammatory stress such as in orthostatic tachycardia syndrome (POTS) (21).

A recent ANS meta-analysis in MS (22) included 16 studies with over 600 patients tested with three or more cardiovascular autonomic tests showed a high rate of cardiovascular dysfunction among patients with MS.

Employing for the first time a clinical bed-side cardiovascular ANS test battery together with classical pupillometry and OCT evaluation of retinal neurodegeneration, our aim was to study early signs of ANS dysfunction and its' relation to neurodegeneration in a newly diagnosed MS population.

## Materials and Methods

### Participants

**Figure 1:**
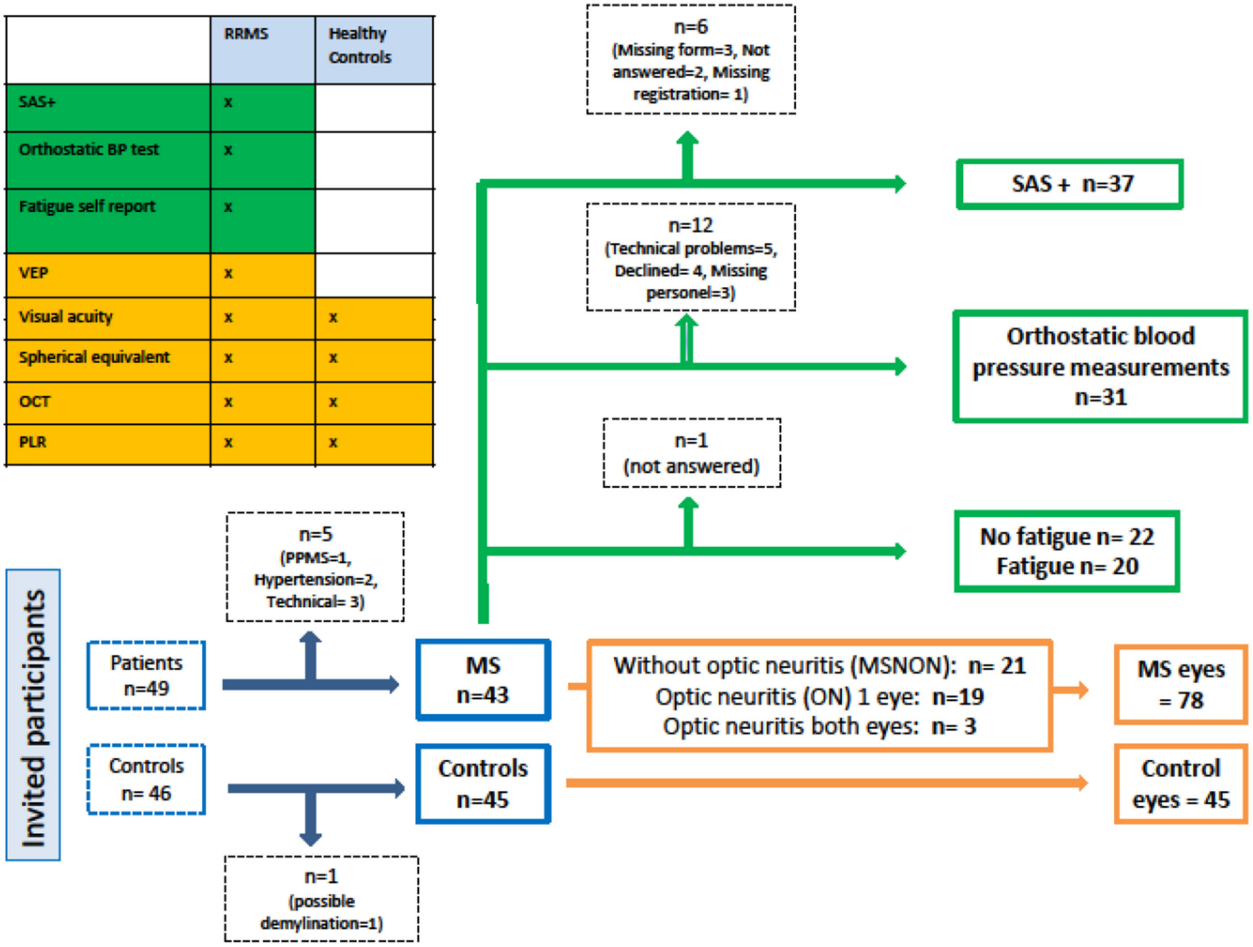
Study flow chart. SAS+, Survey of Autonomic Symptoms and orthostatic symptom scores from the Autonomic Symptom Profile; BP, Blood pressure; VEP, Visual evoked potential; OCT, Optical coherence tomography; PLR, Pupillary light reflex; RRMS, Relapsing remitting multiple sclerosis; PPMS, Primary progressive multiple sclerosis; MS, Multiple sclerosis.

Newly diagnosed relapsing remitting MS (RRMS) patients (*N* = 49), according to the revised McDonald Criteria (2010) (23) and healthy controls (*N* = 46), enrolled in an ongoing longitudinal prospective study on cognition and neuroimaging (24) were invited to participate in this study of autonomic pupillary function in relation to retinal architecture. Forty-three RRMS patients and 45 healthy controls were eligible for analyses. A subset of the included MS patients (*N* = 37) were examined with a set of self-report forms as well as bed-side orthostatic blood pressure (BP) and heart rate tests (*N* = 31). A flow chart of the included participants is presented in Figure 1.

Measurements were randomly conducted throughout the day for both groups. Patients and controls were examined during the same period, but the examiners were not “blinded” regarding the status of the participants.

All participants gave written informed consent and the study was approved by the regional ethical committee of South Eastern Norway (REK 2011/1846 A).

### Neurological and Neuropsychological Examinations

The patients had a complete neurological examination and cerebral magnetic resonance imaging (MRI) performed within 2 weeks of the ophthalmological and pupillary measurements. All patients were clinically stable between the examinations. Grading of neurological disability was assessed using The Expanded Disability Status Score (EDSS) (25). Fatigue was assessed with the Fatigue Severity Scale (FSS) (26). We applied a cut off at FSS > 4 classifying the patients as fatigued.

### Magnetic Resonance Imaging (MRI)

The cerebral MRI scans were performed on the same 1.5 Tesla scanner (Avanto, Siemens Medical, Erlangen, Germany) equipped with a 12-channel head coil. The following sequences were acquired: (1) sagittal 3D FLAIR, (2) pre-contrast sagittal 3D T1 MPRAGE, and (3) post-contrast sagittal 3D T1 MPRAGE started approximately 7 min after the contrast agent injection at a dose 0.2 ml/kg (Dotarem, Laboratoire Guerbet, Paris, France). All scans were evaluated by one neuroradiologist (PS) (blinded to clinical symptoms and findings in the patients) for the presence of brain stem lesions. The location of the lesions was registered (pons or/and medulla oblongata).

### Ophthalmological and Pupillometric Examinations

The patients underwent an ophthalmological examination, including the swinging flashlight test. For all the participants we measured best corrected visual acuity (BCVA) expressed as the logarithm of the minimum angle of resolution (logMAR), in both eyes. Spherical equivalent was calculated and noted in diopters.

The PLR was tested with the Compact Integrated Pupillograph (CIP) version 13.00 from AMTech (Dossenheim, Germany) on both eyes in the patients and randomly on one eye in the healthy controls as far as in 16 who underwent examination of both eyes to balance the number of eyes in the three eye groups as described in the flow chart (Figure 1).

Dark adaptation for 5 min preceded the tests which were conducted with a fixated gaze, but without accommodative cues to avoid confounding pupillary constriction. Measurements were undertaken in darkness with chin and forehead rest in fitted position. The tests were conducted by the first author. When the trigger button was pushed by the examiner a clear yellow visible LED (585 nm) omitted an optical stimulus for 200 ms with an intensity of 784 cd/m^2^ while 2 infrared (880 nm) bluish gray ones illuminated the tested eye and the acquisition of the horizontal pupil diameter was measured with a sampling rate of 250 Hz during 4 s (2 s longer than the default setting from AMTech). The data was directly transferred to a computer with LoOK! software (AmTech) installed for storage and further analyses. Continuous measurement of the pupillary diameter permitted repeated measures of PLR with attended baseline diameter between each measurement. In this study 4 valid measurements were stored and used for further analyses for each included eye.

Various variables describing the function of the autonomic nerves in the eye were calculated according to previous publications (7, 8). LoOK! automatically calculates mean values for the initial diameter (the dark adapted diameter, hereby called the baseline) before each stimulus, the latency from the stimulus is given to the first sign of contraction, the constriction velocity and the amplitude of the contraction. The amplitude and the constriction velocity are dependent on the parasympathetic branch of the PRL. Whereas the 75% re-dilation time and velocity, extracted using a separate software application from AMTech made for this study, is mostly dependent on the sympathetic branch of the PLR.

### Optical Coherence Tomography (OCT)

Optohistologic slicing of the retina was carried out with the spectral domain RS-3000 OCT Retina Scan (Nidek Inc., CA, USA) with a scanning speed of 53,000 A-scans/s and 4 μm digital resolution. The examinations were performed by the first author (SRB). Peripapillary retinal nerve fiber layer thickness (pRNFL) data were obtained with the Disc Circle protocol with a scan width of 3.45 mm centered on the optic nerve head without crossing of the two inner scan circles. The ganglion cell layer (GCL+IPL) thickness measurements were automatically generated from the 9 × 9 mm macula map scan glaucoma segmentation in the included software. All scans included had a signal strength of 8/10 or better.

### Visual Evoked Potentials (VEP)

Visual evoked potentials (VEPs) delay to P100 were obtained as described previously (27) and were evaluated by two experienced clinical neurophysiologists (KN and LE). According to the laboratory-specific reference values, VEP was regarded as pathological with a P100 latency of more than 110 ms and/or with a difference in delay of at least 6 ms between the eyes.

### Autonomic Self-Reports and Bed Side Orthostatic Testing

The patients conducted the self-report Survey of Autonomic Symptoms (SAS) questionnaire (28) consisting of 11 items in women and 12 in men. SAS aims to discover mild autonomic neuropathic symptoms. It is brief and easy to score, but has not previously been tested in early MS patients. We also used questions from the Autonomic Symptom Profile (ASP) (29) covering orthostatic symptoms with timeline. The SAS questionnaire and the ASP questions have been properly translated (and back translated) to Norwegian for another study (30).

As described in the original article of Zilliox et al. the patients were grouped according to SAS-score > 3, while the total impact score (TIS) cut off was > 7. A 4 points' cut-off as described by Suarez et al. at, was used to define orthostatic intolerance. The orthostatic sub-score provided by ASP both graded the complaints and their duration.

Bedside orthostatic testing was performed by the first author the same day as the ophthalmological examinations. The examination room was normally lit, with regulated heating and air conditioning and without any surrounding disturbances. BP and heart rate measurements were conducted automatically at preset intervals with a Dinamap procare V100 (GE Healthcare). Baseline was established with the participant comfortably in supine position for 10 min before asked to stand up for 5 min to calculate orthostatic mean values after 2, 3, 4, and 5 min of standing. The participants were then asked to lay down again for another 2 min for repeated measurements.

### Statistical Analyses

SPSS version 24, Chicago, IL was used for statistical analyses. Independent samples *t*-test were used to test for differences between patients and controls. Linear regression analyses were used to assess the association between signs of retinal neurodegeneration and disease duration. A significance level of 0.05 was applied for all analyses. Levene's test was used to assess homogeneity of the population variances. A Pearson correlation coefficient was produced to assess the relationship between variables.

## Results

### Ophthalmic and PLR Group Characteristics

Newly diagnosed patients with low EDSS and healthy controls were matched on age and gender as summarized in Table 1. Time since diagnosis is of shorter duration than disease duration and is due to the fact that patients often have had symptoms in concordance with MS for some time when the diagnosis is made. Half of the patient group presented with a history of optic neuritis, in all patients more than 3 months prior to the examinations. Brain stem lesions on MRI were visible in 56%.

**Table 1:** Group characteristics of patients and controls.

	**Patients (*n* = 43)**	**Controls (*n* = 45)**	***P* value**
Age, years, mean (SD, range)	34.23 (6.96, 21–49)	33.20 (6.91, 21–46)	0.487
Gender, female, *n* (%)	31 (72)	31 (69)	0.884
Neurological disability, EDSS, mean (SD, range)	1.88 (0.87, 0.0–4.0)	n.a.	
Time since diagnosis, years, mean (SD, range)	1.47 (1.0, 0.08–3.58)	n.a.	
Disease duration, years, mean (SD, range)	2.02 (2.53, 0.33–10.67)	n.a.	
Optic neuritis, *n* (%)	22 (51)	n.a.	
Presence of brain stem lesions on MRI, *n* (%)	24 (56)	n.a.	

*EDSS, The Expanded Disability Status Score; SD, Standard deviation; MRI, Magnetic resonance imaging*.

The ophthalmic characteristics of all the examined eyes are summarized in Table 2. Eyes with prior optic neuritis, confirmed with the presence of prolonged VEP latency to P100, differed from the two other groups concerning retinal architecture and latency of the PLR.

**Table 2:** Characteristics of eyes with (ON eyes) and without previous optic neuritis (MSNON) and eyes of healthy controls.

	**ON eyes (*n* = 23)**	**MSNON eyes (*n* = 55)**	**Control (*n* = 45)**
Spherical equivalent, in diopters, mean (SD)	−0.91 (1.17)	−0.69 (1.57)	−0.79 (1.66)
Best corrected visual acuity in logMar, mean (SD)	−0.02 (0.10)	−0.07 (0.08)	−0.09 (0.01)
pRNFL in μm, mean (SD)	92.90 (12.86)	101.17 (10.94)	104.33 (12.18)
tRNFL in μm, mean (SD)	60.35 (17.37)	68.13 (13.78)	68.16 (11.53)
Ganglion cell layer + inner plexiform layer, superior half, μm (SD)	91.80 (13.49)	101.85 (8.68)	105.96 (8.20)
Ganglion cell layer + inner plexiform layer, inferior half in μm, mean (SD)	93.25 (11.39)	102.72 (7.91)	107.22 (8.12)
PLR baseline in mm, mean (SD)	6.42 (0.82)	6.50 (1.01)	6.60 (0.96)
Pupillary light reflex latency in ms, mean (SD)	251.50 (16.21)	240.28 (13.83)	239.69 (15.68)
Latency p100 in ms, mean (SD)	118.59 (10.56)	104.00 (4.33)	–

*pRNFL, Peri-papillar retinal nerve fiber layer; tRNFL, Temporal section of peri-papillar retinal nerve fiber layer; PLR, Pupillary light reflex*.

For further analyses we therefore used one asymptomatic eye from the patients and one eye from the healthy controls to study the link between neurodegeneration and dysautonomia.

Table 3 presents a comparison of MSNON eyes compared to the healthy controls, one eye from each participant. It shows no difference in BCVA or in spherical equivalent between patients and controls. There was a difference in the ganglion cell layer thickness in the MSNON eyes compared with the healthy controls. There was no difference in the peripapillary nerve fiber layer (pRNFL) or in the PLR latency. Patients and healthy controls were not different concerning PLR measures of parasympathetic and sympathetic function.

**Table 3:** Eye characteristics of MS patients without previous optic neuritis (MSNON) and the healthy controls.

	**MSNON (*n* = 38)**	**Control (*n* = 45)**	***p*-value**
Spherical equivalent in diopters, mean (SD)	−0.82 (1.58)	−0.79 (1.66)	0.94
Best corrected visual acuity in LogMar, mean (SD)	−0.07 (0.09)	−0.08 (0.09)	0.65
pRNFL in μm, mean (SD)	100.24 (10.72)	104.33 (12.18)	0.11
tRNFL in μm, mean (SD)	67.26 (13.32)	68.16 (11.53)	0.74
Mean ganglion cell layer + inner plexiform layer in μm, mean (SD)	102.30 (7.87)	106.59 (8.02)	0.02
PLR baseline in mm, mean (SD)	6.54 (0.97)	6.60 (0.96)	0.78
PLR latency in ms, mean (SD)	240.87 (13.46)	239.69 (15.68)	0.71
Constriction velocity in mm/s, mean (SD)	5.7 (0.69)	5.50 (0.77)	0.40
Time to minimum diameter, s, mean (SD)	0.84 (0.06)	0.83 (0.08)	0.74
75% redilatation velocity in mm/s, mean (SD)^*^	2.80 (1.55)	3.15 (1.87)	0.82
75% redilatation time in mm, mean (SD)^*^	2.64 (0.51)	2.68 (0.62)	0.48

* *MSNON, n = 32, and controls, n = 41. Pupillary light reflex = PLR. pRNFL, Peri-papillar retinal nerve fiber layer; tRNFL, Temporal section of peri-papillar retinal nerve fiber layer; PLR, Pupillary light reflex*.

**Figure 2:**
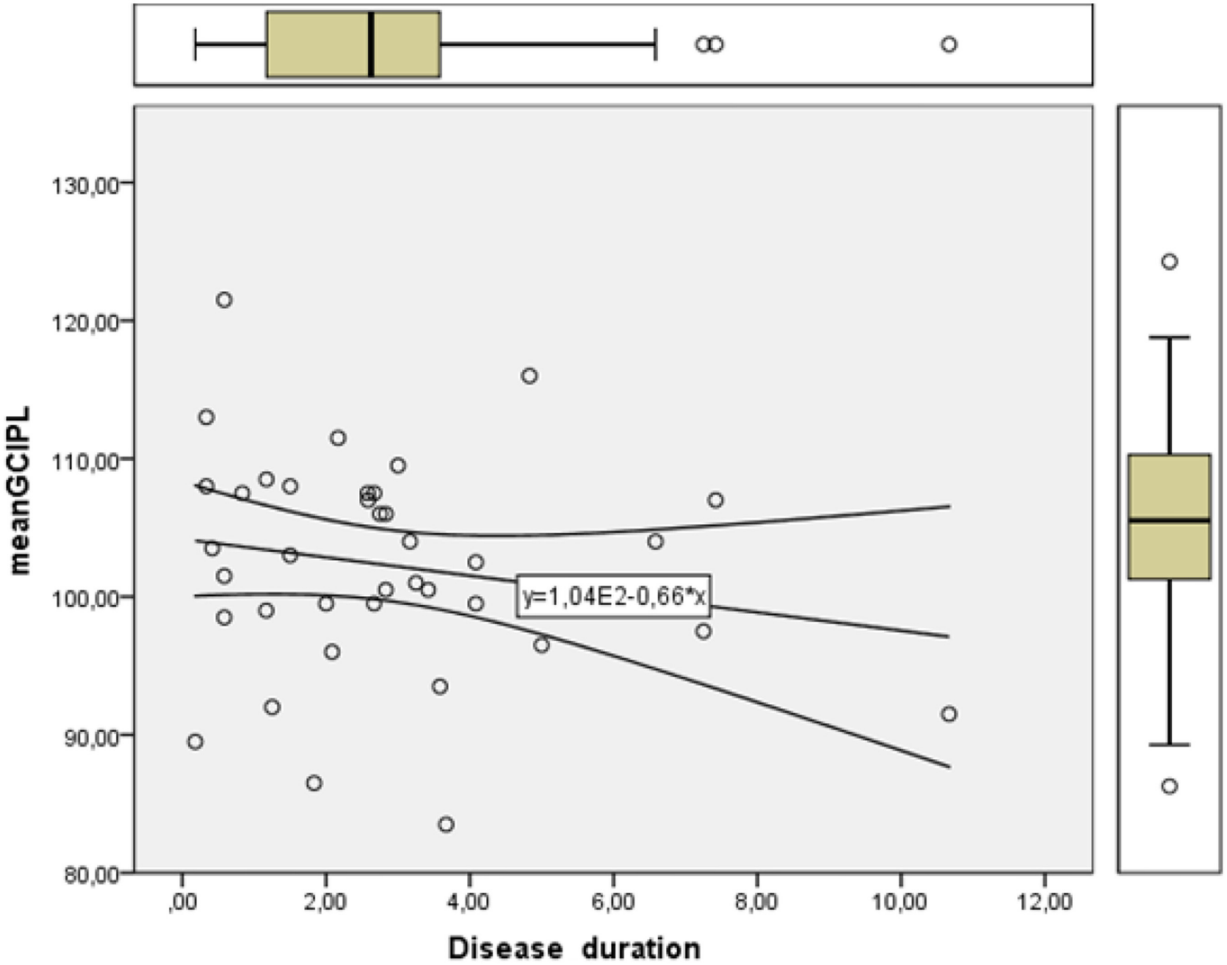
Linearity plot of retinal ganglion cell layer thickness in μm (mean GCIPL) measured with optical coherence tomography showing a gradual reduction with increasing disease duration in years.

Linear regression showed lower mean GCIPL with longer disease duration as presented in Figure 2.

### Autonomic Bed Side Orthostatic Testing

Results of the bed side orthostatic testing are presented in Table 4.

**Table 4:** Orthostatic bed side tests.

	**Baseline**		**Standing**			
			**Mean of 2 and 3 min standing**		**Mean of 4 and 5 min standing**	
	**Mean BP (SD), mmHg**	**Mean HR (SD), per min**	**Mean BP (SD), mmHg**	**Mean HR, (SD) per min**	**Mean BP (SD), mmHg**	**Mean HR (SD), per min**
All included patients (*n* = 31)	109 (8)/64 (6)	58 (8)	112 (11)/69 (8)	76 (12)	113 (9)/69 (8)	76 (13)
HR increase ≥30 beats/min (*n* = 7)	110 (8)/64 (5)	57 (11)	112 (9)/70 (7)	88 (10)	113 (6)/70 (8)	90 (11)

*BP, Blood pressure; HR, Heart rate*.

Seven patients (23%) presented test results compatible with postural orthostatic tachycardia syndrome (POTS) with sustained heart rate increase of ≥30 beats/min within the first 5 min of standing and without signs of orthostatic hypotension. Three of those reported having had symptoms for a longer period than 3 months fulfilling the diagnostic criteria for POTS (31). The patients with POTS had a mean age = 30.3 years (range 29–33); mean EDSS = 1.3 (range 1.0–1.5), and a mean FSS = 2.7 (range 1.3–4.7) representing 10% of the test group.

### Self–Reports

ANS, orthostatic and fatigue symptoms are presented in Table 5. Upon subgrouping, according to standardized cut-off values (28) our patient group showed that 43% reported symptoms of autonomic dysfunction (SAS ≥ 3) and 32% had pronounced symptoms (TIS ≥ 7).

**Table 5:** Patients self-reported autonomic symptoms and fatigue.

		**MS, *n* (%)**	**Mean score (SD)**
Survey of Autonomic Symptoms (= SAS)		37	2.2 (1.4)
	SAS score of <3 points	21 (57)	1.1 (0.7)
	SAS score of 3 or more points	16 (43)	3.6 (0.5)
Total impact score (= TIS)		37	5.3 (4.1)
	TIS score of <7 points	25 (67)	2.9 (1.9)
	TIS score of 7 or more points	12 (32)	10.3 (2.6)
Orthostatic subscore		36	4.3 (2.5)
Fatigue Self Report (FSS)		41	3.8 (1.9)
	FSS score of <4 points	23 (55)	2.6 (0.9)
	FSS score of 4 or more points	18 (45)	5.3 (0.9)

The patients presented a mean score above 4 on the orthostatic questionnaire, revealing a significant level of symptoms. We also discovered that almost half of the patients (45%) reported a high level of fatigue with FSS ≥ 4 based on the fatigue questionnaire.

Correlation analysis between FSS and SAS showed a Pearson coefficient of 0.44 (*r* = 0.044, *n* = 42, *p* = 0.004). There were no significant differences in the mean FSS scores between patients with or without ANS symptoms.

### Test Battery

Of the 31 patients who undertook all the tests in our study no one presented with findings on the PLR test. However, 74% reported significant findings on the self-report questionnaire SAS+, and 23% had signs of orthostatic dysfunction on bed-side testing. As shown in Table 6 16% were positive on these latter two tests.

**Table 6:** The total of patients with findings either on the autonomic symptom questionnaire, the orthostatic bed-side test, or testing of the pupillary light reflex.

	**SAS+, *n* (%)**	**Bed side test with HR ≥ 30 beats/min, *n* (%)**	**PLR, *n* (%)**	**SAS+ AND Bed side test with HR ≥ 30 beats/min, *n* (%)**
Patients participating in all the tests *n* = 31	23 (74)	7 (23)	0 (0)	5 (16%)

*SAS+, Survey of Autonomic Symptoms together with orthostatic symptom scores from the Autonomic Symptom Profile; PLR, Pupillary light reflex*.

## Discussion

We found a significant difference in mean GCIPL in MSNON eyes compared to matched control eyes. Mean GCIPL showed as expected a negative linearity with increasing disease duration. There was no difference in mean pRNFL between MSNON and healthy controls. If there is a primary retinal neurodegeneration with a functional correlate to pick-up on early it is in the macula where our visual acuity is situated and the ganglioncell density is the highest that one would be able to detect it first. Secondary degeneration of the axons from these dead neurons would also occur and possibly be measured as a loss of pRNFL on OCT, especially in the temporal region where the axons from the papillomacular bundle lies and the axons are the thinnest and most vulnerable. An ongoing loss of retinal ganglion cells without any previous clinical signs of inflammatory relapses or significant loss of retinal axons depicted with the peripapillary ring scan on OCT is described in MS (15), and confirmed by the present study. Nonetheless our results underline the premature emergence of retinal neurodegeneration. Longitudinal studies of patients diagnosed with clinically isolated syndrome may confirm if this neuronal loss is an onset feature of MS.

Half of the patients presented with ANS symptoms and a high level of fatigue in line with previous reports (2, 32). We found a medium level of correlation between the SAS score and the FSS score and no significant difference between patients with or without ANS symptoms. Self-reports are assessments of self-perceived health status often designed for population studies. The choice of tests is important to ensure its' relevance and proven efficacy regarding what we are searching for and in our case there are few tests that cover ANS symptoms in early MS and publications are scares. One might object to the use of self-reports on the basis of subjectivity since patients might over rate their symptomatic burden and they may also find the tests tiresome and become biased toward their inner feeling of distress produced by the fact that we ask them to complete the questionnaire.

**Figure 3:**
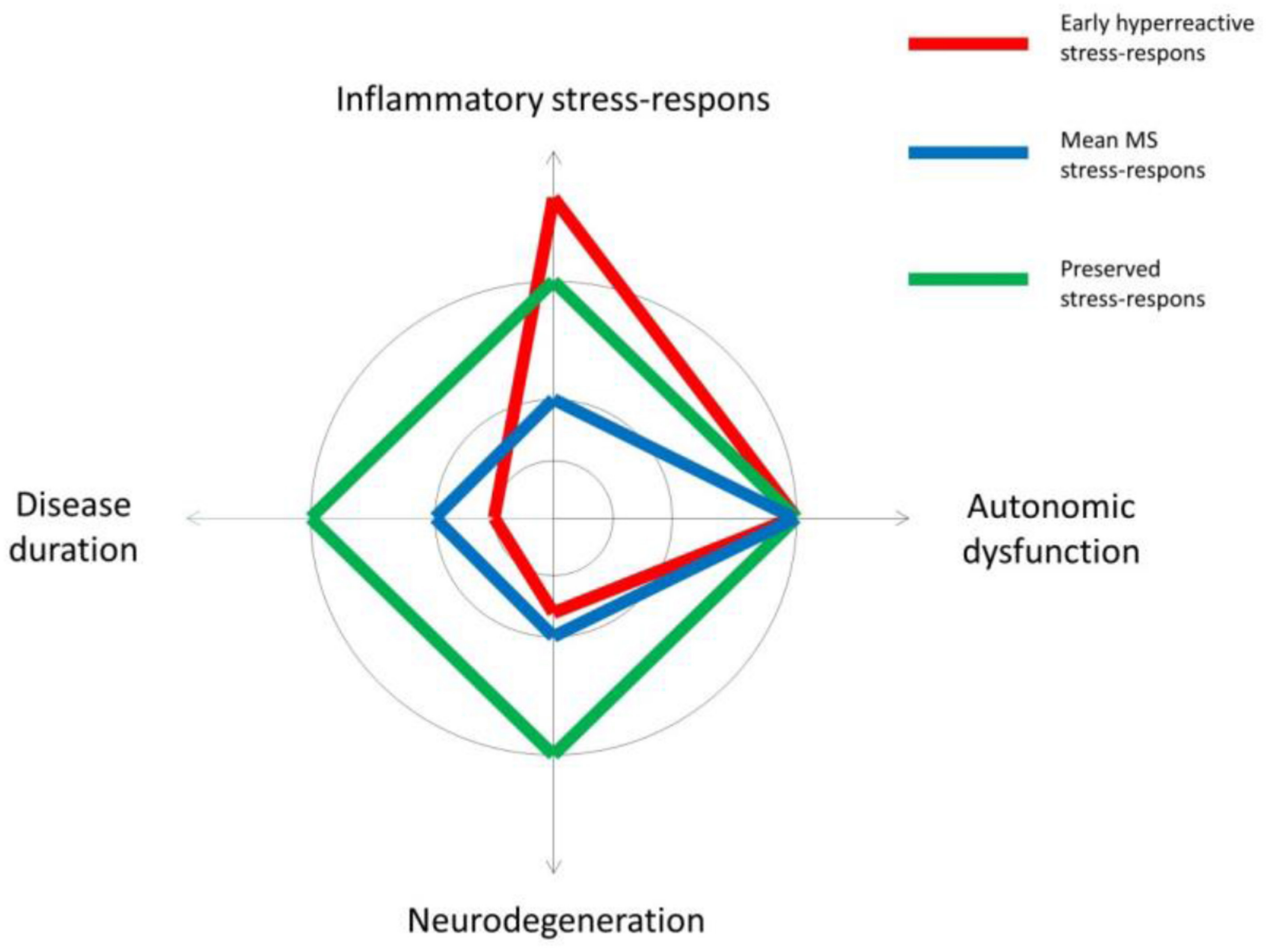
A theoretical illustration of different profiles of neurodegeneration in relation to symptoms of autonomic dysfunction and the inflammatory stress-response in early multiple sclerosis (MS).

Even though we did not find classic orthostatic hypo-or hypertension, 23% of the MS patients presented a high HR response upon bed side testing. Three patients (10%) fulfilled the criteria (33) of POTS in line with the few prior MS studies on this field (34, 35). Our patients with POTS tended to be young patients with a low EDSS score, only one had fatigue. The diagnostic recognition of this entity is important in order not only to treat the symptoms and to help patients resume their regular daily activities and work life, but it is also in line with the hypothesis of an altered stress response in MS (36) occurring early in the disease course. As described in a recent review “the onset of POTS is typically precipitated by immunological stressors” (21), this underlines the fact that POTS patients often present with positive autoimmune tests and immune system dysfunction. Cardiovascular autoantibodies with a causative role have been proposed in POTS, but another hypothesis is the presence of abnormally increased sympathetic activity alongside findings of peripheral autonomic neuropathy. Studies of cardiovascular risk in MS support this latter theory with upregulation of the sympathetic stress response early in the disease course (37). However, the present findings do not indicate that peripheral neuropathy can explain the POTS findings among our patients. A schematic illustration of different profiles of neurodegeneration, ANS symptoms and stress-response in early MS is proposed in Figure 3. As this theoretical scheme underlines, we are faced with changes in highly adaptive and complex systems when studying the central nervous and the immune system. The finely tuned communication between these systems in healthy subjects, is disrupted and probably a highly pathogenic factor in MS due to an excess of inflammatory processes that may be further amplified by coexisting psychological distress (33).

The sympatho-adrenergic branch of the ANS is particularly important in the cross-talk between the immune system and the CNS. Both adrenalin and noradrenaline are synthesized from dopamine which has known immune effects (38). Hypothetically for some MS patients an initial overshooting sympathetic upregulation caused by inflammatory stress may be followed by a negative feed-back loop that counteracts the innate immunosuppressive effect of adrenaline and noradrenaline. Hence the immune cell driven neurodegeneration in the CNS in MS is in these cases free to expand itself. Dopamine on the other hand is synthesized from phenylalanine through several steps and metabolomics have showed diminished levels of phenylalanine in the cerebrospinal fluid of MS patients (39), this could both counteract the innate immunosuppressive effect and alter the ANS output. Further studies focusing on the connection between ANS dysfunction, dopamine and phenylalanine levels could give insight into what causes the cardiovascular and neurodegenerative interplay in MS.

This study employed bedside tests with equipment available in most hospital settings together with a classical research tool, the pupilometer, in order to search for a link between early signs of neurodegeneration and ANS dysregulation in MS. The bedside tests are designed to discover orthostatism and POTS related to alterations in ANS regulation of cardiovascular function. Our test battery would have been more sensitive and complete regarding early cardiovascular ANS changes if supplemented with a Valsalva maneuver test and continuous BP measurements (40). Previous PLR studies (4, 10, 11, 41) have mostly studied patients with advanced stages of MS and a possibly much higher degree of retinal ganglion cell and axonal loss. However, this is the first time OCT is used in conjunction with an ANS test battery consisting of other test than PLR measurements.

There was no difference in the retinal nerve fiber layer depicted with the peripapillar ring scan on OCT or in the PLR latency between MSNON and healthy controls. Changes in PLR measures could then have been interpreted as alterations in ANS branches of the PLR since the optic nerve had no signs of disease affection. We did not find any changes in the PLR comparing patients and controls in this study, underlining the robustness of the eye pupil as a proxy of function and usability in other settings as for instance as our group has published earlier, in cognitive testing in MS (27).

Additionally the melanopsin-expressing retinal ganglion cells influencing the PLR are more resistant to injury like optic neuritis than other ganglion cells (42) and this adds to the fact that small structural retinal changes may not translate into PLR changes.

Our study is limited by the small subgroup numbers. Further testing of the concept of altered stress response in a larger group of patients is warranted. A study design where both patients and controls are tested with all the tests is advisable in order to expand the normal reference standards. Health related quality of life scales would also add value to our self-reports repertoire. Another future aim is to establish robust structure –function correlates of retinal changes and PLR measurements, in other words at what level PLR results reflect structural retinal neurodegeneration and not ANS changes. Longitudinal cohorts may give the answer. PLR testing in the blue light spectrum of 400–500 nanometer (43) may disentangle at what level a possible loss of melanopsin containing ganglion cells affects ANS regulation of the pupil through input from the retina. Another interesting field in CNS and ANS cross-talk is the concept of interoception, the sensing of bodily signals, which is shown to be altered in MS (44) and should be considered in future MS ANS study design.

The autonomic test battery in our study is robust. We tested for ANS dysfunction and neurodegeneration employing the same input organ, the eye. Nevertheless, future studies may consider using a more extensive battery of ANS tests. We note that autonomic testing is a difficult domain and believe that establishment of screening tests batteries in the clinic will provide answers to the questions our patients are struggling with every day.

In conclusion, our study unveiled retinal neurodegeneration in asymptomatic eyes in newly diagnosed MS patients with a high frequency of fatigue and a high prevalence of ANS symptoms. Several of the patients had symptoms and signs consistent with POTS. The possible interplay between presymptomatic neurodegeneration, fatigue, and symptoms of autonomic dysfunction needs further investigation.

## Ethics Statement

All participants gave written informed consent and the study was approved by the regional ethical committee of South Eastern Norway (REK 2011/1846 A).

## Author Contributions

SdRB wrote the manuscript, conducted the ophthalmological, pupillometric, optical coherence tomographic, and bed-side autonomic examinations as well as the statistical analyses. GN planned the study, conducted the neurological examinations, administered, and gathered all the self-report data and provided data for Table 1. KN contributed with study planning, analyzes of the VEPs and valuable input on how to interpret the autonomic findings and structuring of the manuscript. LE analyzed the VEPs. PS performed the MRI analyzes. MW-H conducted the neurological examinations. HH, LD, EK, and BL contributed with research planning and supervising the study. EC was the main supervisor of this study and gave valuable input throughout the whole process including statistical analyzes as well as structuring and writing of the manuscript. All authors reviewed and approved the final manuscript.

### Conflict of Interest Statement

The authors declare that the research was conducted in the absence of any commercial or financial relationships that could be construed as a potential conflict of interest.
